# Improved 3D Printing and Cell Biology Characterization of Inorganic-Filler Containing Alginate-Based Composites for Bone Regeneration: Particle Shape and Effective Surface Area Are the Dominant Factors for Printing Performance

**DOI:** 10.3390/ijms23094750

**Published:** 2022-04-26

**Authors:** Vera Bednarzig, Stefan Schrüfer, Tom C. Schneider, Dirk W. Schubert, Rainer Detsch, Aldo R. Boccaccini

**Affiliations:** 1 Department of Materials Science and Engineering, Institute of Biomaterials, University of Erlangen-Nuremberg, 91058 Erlangen, Germany; vera.bednarzig@fau.de (V.B.); tom.c.schneider@fau.de (T.C.S.); 2 Department of Materials Science and Engineering, Institute of Polymers, University of Erlangen-Nuremberg, 91058 Erlangen, Germany; stefan.schruefer@fau.de (S.S.); dirk.schubert@fau.de (D.W.S.)

**Keywords:** 3D printing, alginate, bioactive glasses, rheology, organic–inorganic composites

## Abstract

The use of organic–inorganic 3D printed composites with enhanced properties in biomedical applications continues to increase. The present study focuses on the development of 3D printed alginate-based composites incorporating inorganic fillers with different shapes (angular and round), for bone regeneration. Reactive fillers (bioactive glass 13–93 and hydroxyapatite) and non-reactive fillers (inert soda–lime glass) were investigated. Rheological studies and the characterization of various extrusion-based parameters, including material throughput, printability, shape fidelity and filament fusion, were carried out to identify the parameters dominating the printing process. It was shown that the effective surface area of the filler particle has the highest impact on the printing behavior, while the filler reactivity presents a side aspect. Composites with angular particle morphologies showed the same high resolution during the printing process, almost independent from their reactivity, while composites with comparable amounts of round filler particles lacked stackability after printing. Further, it could be shown that a higher effective surface area of the particles can circumvent the need for a higher filler content for obtaining convincing printing results. In addition, it was proven that, by changing the particle shape, the critical filler content for the obtained adequate printability can be altered. Preliminary in vitro biocompatibility investigations were carried out with the bioactive glass containing ink. The 3D printed ink, forming an interconnected porous scaffold, was analyzed regarding its biocompatibility in direct or indirect contact with the pre-osteoblast cell line MC3T3-E1. Both kinds of cell tests showed increased viability and a high rate of proliferation, with complete coverage of the 3D scaffolds’ surface already after 7 d post cell-seeding.

## 1. Introduction

The need for clinical surgery especially in the field of bone regeneration, restoration and replacement is increasing due to the aging of the world population [1]. This makes human bone one of the most extensively affected and transplanted tissues [2,3]. High success can be achieved with autografts or allografts. Nevertheless, limitations in sources and tissues, high costs, rate of rejection, as well as donor-site morbidity and pain [3,4], make it necessary to find alternative methods of treatment [5]. Bone regeneration approaches based on the use of 3D biomaterial grafts and scaffolds appear as effective alternatives to the use of autographs and allographs. Additive manufacturing (AM) is an attractive method to generate bone grafts and scaffolds from engineered biomaterials. With this layer-by-layer process, even complex 3D structures can be generated mimicking the natural bone structure [6]. Especially with the 3D extrusion technique, printing materials such as polymers, ceramics, glasses and composites thereof is possible, making it the perfect technique to fabricate tailor-made scaffolds for bone regeneration [5,7,8]. The resulting 3D structures can then be used as temporary scaffolds guiding bone regeneration and improving the healing process [3]. 

Jones et al. [3] further mentioned that, to adapt the graft to the patient’s needs in surgery, not only factors regarding the mimicking of natural bone structure are important, but further the manageability of the material and its workability during the operation are paramount. Alginate, as a natural polymer, meets the above criteria, being a highly used and extensively studied biomaterial, finding applications in wound healing, tissue engineering and bone regeneration. Alginate offers high biocompatibility, non-toxicity and the ability of gel formation while being highly cost effective [9,10,11]. With bivalent ions such as calcium (Ca^2+^), alginate can further be crosslinked forming a water insoluble hydrogel [11,12]. Due to their inert character, alginate hydrogels do not offer cell-binding sites and exhibit poor mechanical properties [13,14], being delicate in handling, which limits their usability. To overcome these drawbacks, composites can be exploited, combining alginate with inorganic bioactive particles or fillers [9,15]. For the generation of a 3D interconnected pore system with a defined pore structure by 3D printing without collapsing of the material [16], the used material has to be easily 3D printable based on its rheological behavior, offering high reproducibility and stable printed strands. The improvement of mechanical properties of biopolymers by developing composites is in the focus of different fields of research [17,18]. Here, a wide range of fillers is used to enhance relevant material properties such as the Youngs’ modulus and fracture toughness [19,20,21]. One-step approaches that use hydrogel–filler composites for 3D printing have emerged, where the inorganic filler is directly incorporated into the ink (hydrogel) to alter the rheological properties for improved shape fidelity or cell proliferation and differentiation [22].

One crucial limitation of the size of used fillers is defined by the used needle during printing, which usually ranges between 300 and 600 µm. Too large filler particle sizes or high concentrations can lead to nozzle clogging, therefore rendering the composite ink unsuitable for reproducible 3D printing. Commonly used filler materials include cellulose nanofibers [23,24] or inorganic filler particles such as bioactive glass (BG) [25,26], hydroxyapatite [27] or metallic particles such as gold nano particles [28]. Alginate composites have already shown promising results in combination with inorganic components such as hydroxyapatite, bioactive glasses or other calcium phosphates [9,15,29], being frequently used materials for bone regeneration [3]. For instance, Luo et al. [15] successfully used a combination of alginate, gelatin and hydroxyapatite generating 3D printed scaffolds with an interconnected pore system, being potentially useful for bone regeneration. Other approaches such as those of Aihemati et al. [30], Hung et al. [31] and Hashimi et al. [32] utilize thermoplastic matrix materials combined with inorganic filler particles to print scaffolds via material extrusion for bone regeneration. Commonly used thermoplastics are PLA (poly-lactide acid) and PCL (poly-caprolactone). However, due to the nature of thermoplastic FDM printing, it is not possible to integrate cells into the printing process [33]. There are also other AM techniques that can be used for the generation of bone scaffolds, namely photopolymerization (SLA), binder jetting, powder bed fusion and direct energy deposition [34]. The latter three options are mainly used to process metals or ceramics. However, the mentioned techniques often require either expensive hardware or are very limited in terms of usable materials. Therefore, we chose material extrusion printing for the present study.

The main subject of interest is usually the correlation of infill percentage to printing results, mechanical properties, and cell differentiation. Other investigations were set on the rheological properties, printability and upcoming forces at the printing nozzle during 3D printing [16,35,36,37,38]. 

Surprisingly, the influence of the filler particle characteristics on the rheological properties of the ink, printing performance and on the resulting 3D printed object is often overlooked.

Therefore, the aim of this research was focused on 3D printing alginate-based composite inks, incorporating a range of round and angular fillers in micrometer particle size. In addition, the difference between active and inert filler components using inert glass, BG and hydroxyapatite particles, was considered to investigate their impact on the printing process. The influence of the particle geometry and bioactivity of fillers on the rheological properties and printing results of alginate-based inks was systematically analyzed.

## 2. Results

### 2.1. Particle Size Distribution and SEM Imaging

**Figure 1:**
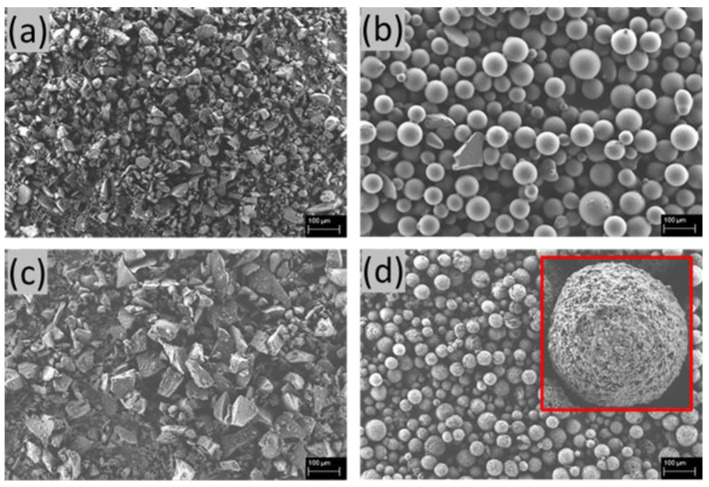
SEM images of the used filler particles; (**a**): angular inert glass, (**b**): round inert glass, (**c**): angular BG, (**d**): round hydroxyapatite. Scale bar: 100 µm. Red square: zoomed in structure of hydroxyapatite particles, exhibiting a rough surface (not observed on the glass particles).

**Figure A1:**
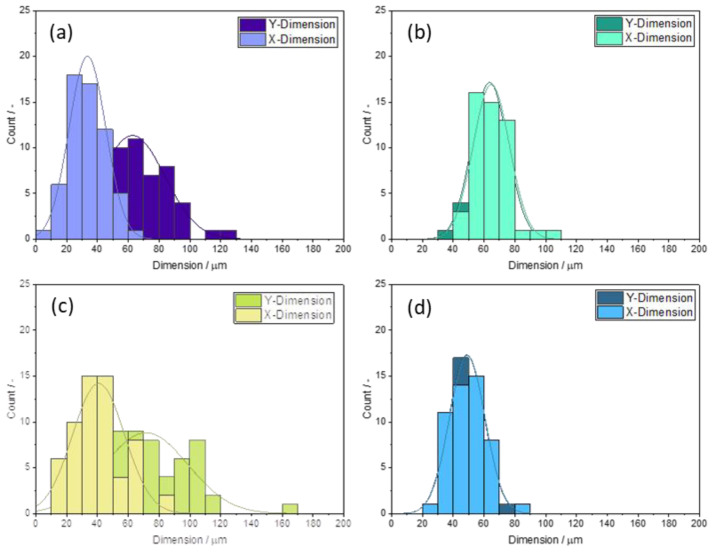
Particle size distribution of the used filler materials: (**a**) angular inert glass, (**b**) round inert glass, (**c**) angular bioactive glass, (**d**) round hydroxyapatite.

Exemplary SEM images for each filler type are shown in Figure 1: angular inert glass, round inert glass, angular BG particles and round hydroxyapatite (HA). The geometrical differences between angular and round fillers are clearly visible. The important optical difference that needs to be mentioned is highlighted in red (Figure 1). The HA particles show a highly structured surface, potentially resulting in an increased effective surface area and roughness compared to the smooth, round inert glass particles. Following the initial optical characterization, particle diameters were analyzed and compared. The results are shown in Figure 1. The median values and Y-to-X ratios are summarized in Table 1. The particle diameter in the X direction always represents the longest distance measured in the X plane, while the Y dimension represents the longest distance measurable perpendicular to the X axis. Only completely visible particles were taken into consideration. The measured particle size distributions are shown in the appendix A1 (Figure A1 in Appendix A).

The median particle size values range between 34 ± 12 μm and 72 ± 21 μm, underlining a successful sieving process. Furthermore, the particle size distribution of the different geometry type filler materials (angular and round) is highly comparable. Hence, the Y–X ratios are approximately identical for the individual geometry type. Here, a ratio of one describes either a perfect sphere or a perfect cubic particle. These findings enable an artifact-free discussion of the influence of particle shape and bioactivity on the printability of the tested composites, as intended in this study.

### 2.2. Filler Density and Volumetric Infill

The resulting filler densities and calculated volumetric infills are summarized in Table 2. The aim of this investigation is to reveal potential network formation (percolation) between particles. Furthermore, the effective surface area of the used particles can be estimated. While this is trivial for solid round particles, some framework conditions had to be considered to keep estimations for angular particles both simple and realistic. Therefore, angular particles are treated as simple cuboids. The average from the measured X- and Y-dimension is used as the missing Z-dimension of angular particles. The higher surface to volume ratio for angular particles compared to spherical ones does result in significantly higher estimated surface areas per milliliter. Further, this leads to more than a two-fold increase in the effective surface area, when comparing angular and round particle geometries. However, since the SEM analysis of HA particles showed a nano-scaled surface roughness (Figure 1), which might lead to a significant increase in surface area, a nitrogen sorption analysis was performed.

### 2.3. Nitrogen Sorption Analysis

The nitrogen sorption analysis revealed an effective surface area of 1.38 m^2^/g for HA particles. This leads to a 68-fold increase in the estimated surface area per ml and therefore the highest estimated value of all filler particles used in this study. The exact values can be seen in Table 3.

Nitrogen sorption analysis was also performed on BG and inert glass particles. Due to their almost nonexistent surface roughness and full density, no results could be measured for these fillers.

### 2.4. Material Throughput Analysation for 3D Printing

The composite inks were prepared as explained in Section 4.1. Briefly, alginate powder and filler particles were blended and added to the liquid component under constant stirring at RT. After homogenization by hand and centrifugation, the material was stored at RT for 24 h until usage.

**Figure 2:**
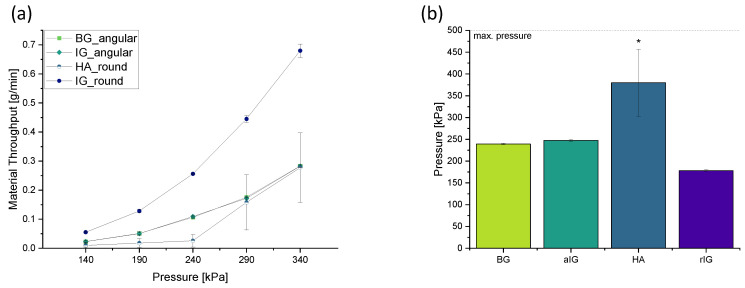
Material throughput of different round and angular particles in an alginate matrix at 5 different pressures, extruded for 1 min (**a**). Comparison of the pressures required to obtain the same material throughput as the reference ink based on angular BG at 240 kPa (**b**). One-way ANOVA statistical analysis indicates significant differences (* *p* < 0.05) of ink HA in relation to all other fillers.

Each material was extruded at five different pressures for one minute. As a reference pressure, 240 kPa was chosen based on initial printing results of the BG-based composite in preliminary studies. Based on this value, the pressure was reduced/increased in 50 kPa steps, two times each (Figure 2a). 

Especially the HA ink presented a significantly different trend compared to the inks with other fillers. A high variation resulting in a high standard deviation of the measured weights of the extruded material, especially at higher pressures, indicated a highly inhomogeneous printing performance of the round HA filler particles in the used HA-alginate composition. During extrusion, the material behaved in a non-linear manner with increasing pressure. Even at high pressures of 290 kPa, temporarily no material could be extruded due to clogging of the nozzle.

BG and aIG inks showed the same trend with negligible standard deviations for all used pressures. No difference was detectable between the two angular filler particles. Both inks double the amount of material being extruded every 50 kPa until 240 kPa. In contrast, round fillers showed a divergent trend, with a significantly lower throughput of ink HA, and a significantly higher throughput of ink rIG compared to the angular filler inks. Unlike ink HA, ink rIG showed a high reproducibility with low standard deviation. The amount of extruded material for ink rIG at least doubles the throughput of inks aIG and BG for all pressures.

As shown in Figure 2b, the necessary pressure to obtain the same throughput (0.106 g/min) of the reference ink (BG) is almost identical for the ink aIG. Only a pressure increase of 5 kPa is needed to obtain the same outcome. Results for ink HA (380 ± 77 kPa) show high variations due to frequent clogging of the nozzle. Since no homogeneous strands could be extruded after 1 min, no definite pressure can be set to generate the same throughput as the reference ink BG. Therefore, comparably high pressures, significantly different from the other compositions, were necessary to dispense the same amount of material, even if a lower pressure would probably be sufficient if needle clogging could be avoided. For ink rIG, a pressure of only 180 ± 2 kPa is needed, which is comparably low especially in contrast to the one used for the reactive counter filler HA. Nevertheless, also rIG ink shows, like inks aIG and BG, a high reproducibility of the pressure values generating the same throughput. Dynamic mechanical analysis was used to validate these findings and generate more detailed insights.

### 2.5. Dynamic Mechanical Analysis (DMA)

**Figure 3:**
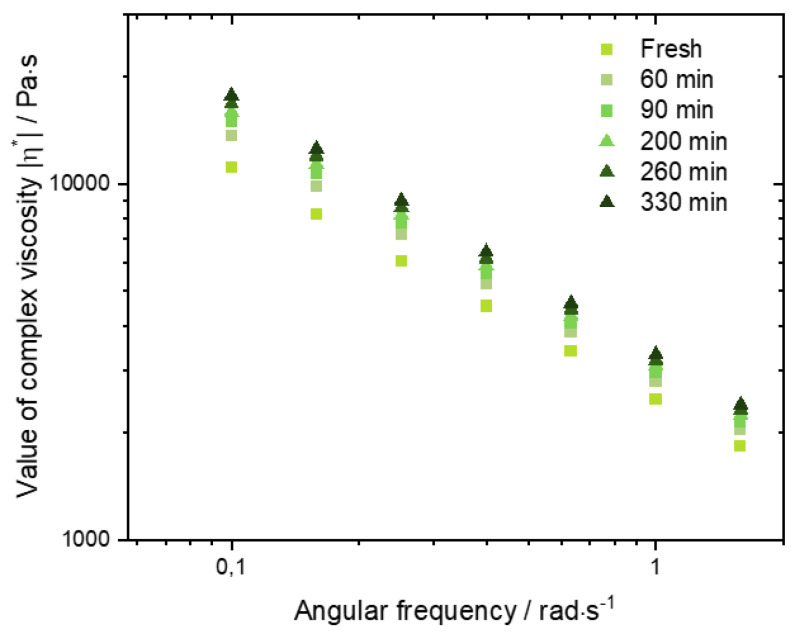
Frequency-dependent value of complex viscosity of a BG–alginate ink in the interval from 0.1 to 1 rad/s for six tested time intervals.

DMA was carried out on the different inks to evaluate the impact of the used filler particle shape and their reactivity on the rheological properties of the inks. Furthermore, the gathered information lays the foundation for obtaining correlations of rheological properties and printing results. The preliminary analysis of the BG composite revealed an increase in the value of complex viscosity at 0.1 rad/s (from 11,062 Pa s to 17,542 Pa s) in 330 min (Figure 3). However, the increase was reduced for every tested time interval. To ensure homogenous rheological properties during the whole printing process a 24 h rest time between sample preparation and characterization was used for all BG inks. This behavior was not observable for any other tested ink and will be therefore not further discussed in this paper.

**Figure 4:**
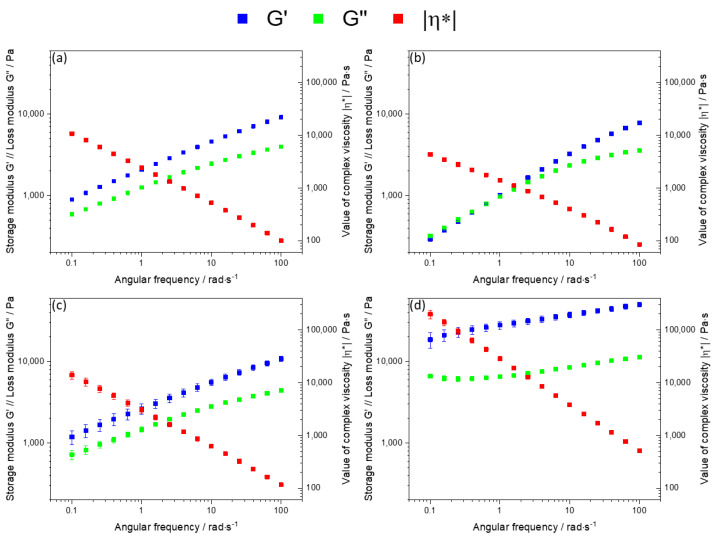
Frequency-sweeps of the tested alginate matrix inks with different fillers; (**a**): angular inert glass, (**b**): round inert glass, (**c**): angular BG, (**d**): round HA.

The rheological properties of all inks were analyzed for obtaining a more detailed correlation between printability and filler type. Here, the focus was on the frequency sweep since flow-type measurements often show measurement artifacts (such as wall slip) for high viscous materials. The averaged results of three measurements for each sample are presented in Figure 4.

The green and blue graphs represent the loss (G″) and storage (G′) moduli. The value of complex viscosity is highlighted in red. A major difference between the tested composites is evident: the sample containing rIG filler shows similar values for G′ and G″ in the low frequency regime with a crossover point at about 1 rad/s. From this point, G′ starts to dominate the viscoelastic material behavior. The value of complex viscosity also shows a slight curvature, hinting towards a more liquid-like material behavior, especially in the low frequency regime. This is not observable for the three other composite inks. Here, G′ always dominates G″, resulting in rheological elastic material behavior in the tested frequency regime. The values of complex viscosity all show power-law behavior.

**Figure 5:**
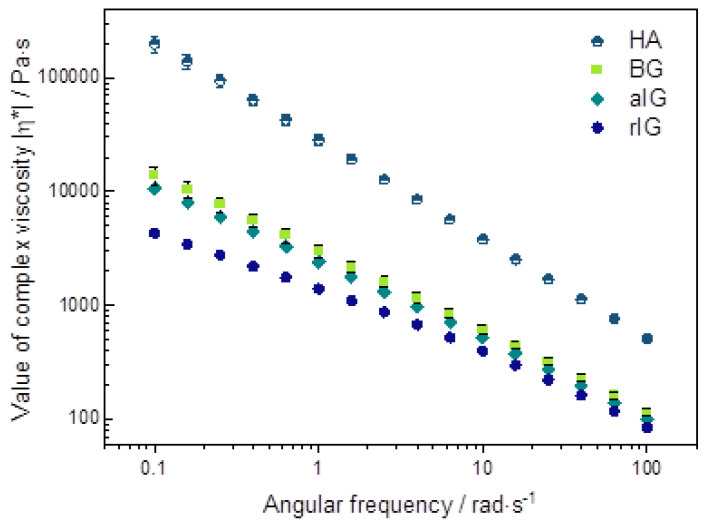
Values of complex viscosities of alginate composite inks in direct comparison of round and angular filler particles incorporated into the alginate matrix.

Furthermore, inks aIG and BG showed comparable material behavior for the storage and loss moduli as well as for the value of complex viscosity, as shown in Figure 5. In contrast, HA filler showed a significant lower influence of the angular frequency on both moduli, resembling a more pronounced gel-like character. Additionally, the value of complex viscosity was shifted by a factor of ten to higher values. In the field of biofabrication, a high value of complex viscosity at low frequencies usually positively influences the shape fidelity [39,40]. Additionally, a rheological solid material behavior reduces strand spreading and improves stackability. Therefore, a prediction of printing shape fidelity from the rheological measurement data results in the following order (from most suitable to deficient): 

HA > BG = aIG > rIG

It has to be mentioned that the presented prediction is only based on the rheological characterization. Other relevant attributes, such as homogeneity of extrusion and nozzle clogging, are not considered and may alter the actual applicability of the inks.

### 2.6. Rheological Modelling

To evaluate the suitability of rheological models, to predict the properties of the inks, the adjusted R^2^ values were determined and compared. Hence, in contrast to the regular statistical R^2^, the number of parameters is taken into consideration. Therefore, over-parameterized models can be identified by decreased adjusted R^2^ values. The results are shown in Table 4.

**Figure 6:**
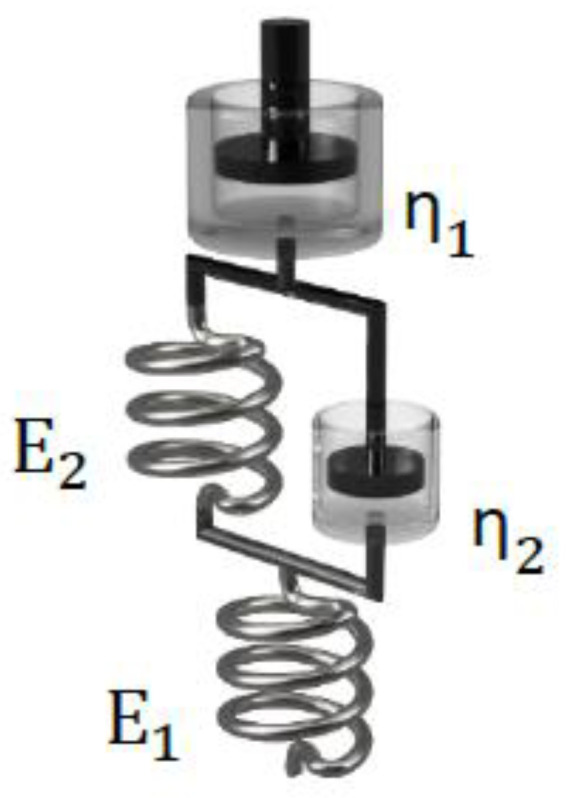
Schematic illustration of the Burgers rheological model.

**Figure 7:**
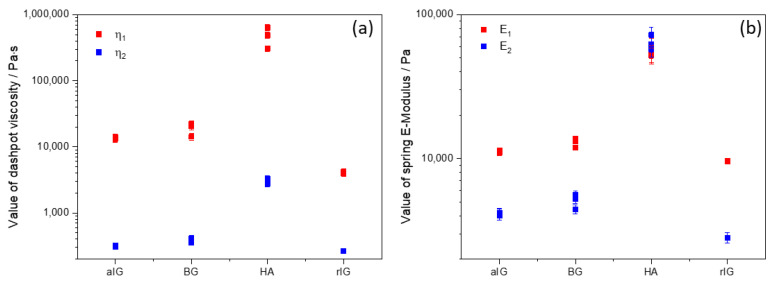
Resulting model parameters for the augmented Burgers model for dashpot viscosity (**a**) and spring YounG′s modulus (**b**) for the different investigated inks.

Since an adjusted R^2^ of one describes a perfect suitability of the used model, it is evident that the augmented Burgers model (further elaborated in [41] and illustrated in Figure 6) is capable to adequately describe all tested samples. A more simple but also suitable model is the Zener model, which can be used to describe viscoelastic solids such as gels or crosslinked polymers [42]. In contrast to the Zener model, the augmented Burgers model features a high viscous Maxwell (or free) dampener. Therefore, a potential flowing (or stress relaxation) of the material for long timescales is covered by this form of the Burgers model. To generate further insights and enable better comparability of the materials, the respective model parameters (dashpot viscosities and spring E-moduli) were determined and are displayed in Figure 7. Here, the index 1 indicates the Maxwell part of the model system, while parameters labeled with the index 2 describe the Kelvin–Voigt part. Error bars indicate the calculated error from error propagation. Previous theoretical [43] and experimental work [41] revealed a correlation of the spreading behavior of hydrogels and the η_1_ parameter of the augmented Burgers model. In our study, inks aIG and BG show similar values for η_1_, while ink rIG shows slightly lower values for all measurements, suggesting more spreading and therefore anticipating lower shape fidelity and stackability of the strands. The respective parameter value for sample HA exceeds all other composites by a factor of 30 and more. Hence, the rheological modelling supports the printability prediction from the experimental rheological analysis presented in the previous section.

When comparing the spring moduli, samples aIG, BG and rIG show similar trends compared to their respective dashpot viscosities. Ink HA however exhibits significantly different behavior. Here, the values for E_1_ and E_2_ are identical within the experimental error. Furthermore, the values of the elastic moduli are increased by a factor of five compared to the remaining inks. 

### 2.7. Printability and Shape Fidelity: Semi-Quantitative Analysis

**Figure 8:**
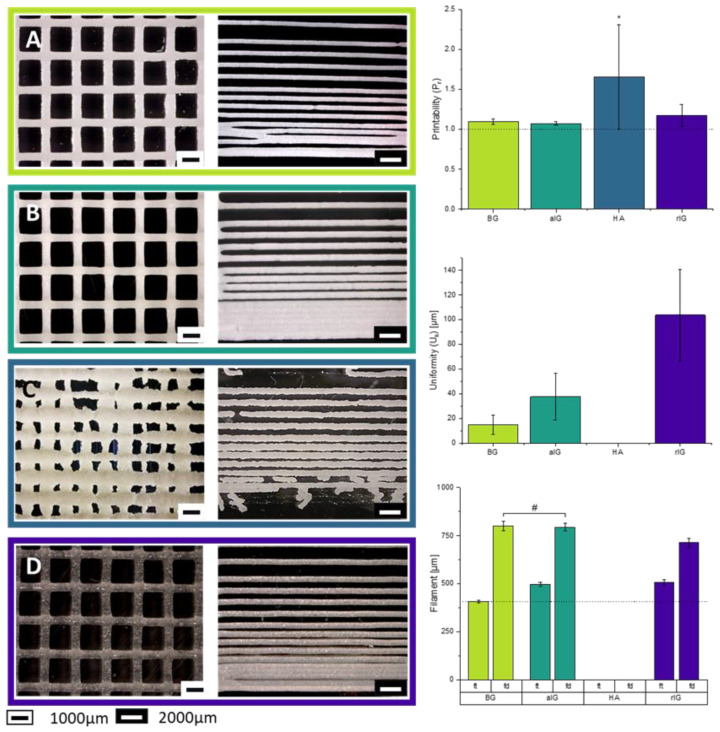
Light microscopy images of printed grids (**left**) and resolution trees (**middle**). (**A**) Ink BG, (**B**) ink aIG, (**C**) ink HA, (**D**) ink rIG. Diagrams show the printing performance results of printability (P_r_), uniformity (U_S_), filament thickness (f_t_) and filament distance (f_d_) (**right**). Statistical analysis using one-way ANOVA indicated a significant difference (* *p* < 0.05) for all samples. Samples labeled with # are found to be an exception.

For the semi-quantitative analysis of the printing behavior, rectangular grids and resolution trees were generated to investigate the influence of the used fillers on the printing performance. Grids with rectangular pores were used for accuracy measurements, including the material’s ability to stay stable in its printed dimension, even if a second layer is printed on top of the first, without shape distortion. Light microscopy images of the printed grids and resolution tress are shown in Figure 8. Further inspection of the printed grids revealed that no phase separation or sedimentation of the filler particles during the composite material fabrication has taken place. Since rheological characterization revealed a relatively high viscosity, such segregation had not been expected.

P_r_ results show no detectable significant difference between inks BG and aIG. Both inks are printable with a high level of reproducibility based on the small standard deviation. Both inks create pores with a P_r_ index of 1.1, with a slightly closer trend to 1.0 and therefore higher accuracy for ink aIG (1.07 ± 0.02). 

Ink rIG (P_r_= 1.17 ± 0.14) with a comparable high standard deviation exhibited less accurate printability, showing flowing of the material and therefore a higher printability index. Even if the rIG ink is printed with the same material throughput, needing 60 kPa less than inks BG or aIG, the material shows less shape fidelity especially in the cross-sections where the two layers overlap, indicating strand merging.

On the other hand, the ink HA displays a P_r_ index of (1.65 ± 0.66) as a result of the incontrollable pores and the loss of shape. Further, based on the frequent clogging of the nozzle, results are not reliable. Depending on the material condition, more, less or even no material was extruded, which is also the case for the resolution tree. Due to this behavior, the P_r_ results for ink HA are just shown for this composition and no further analysis was conducted. Nevertheless, as already mentioned in Section 2.5, if needle clogging for this ink could be avoided, promising results regarding stackability and therefore the generation of interconnected scaffolds could be obtained if the composition of the ink HA would be adapted.

Uniformity (U_s_) results endorse the ones of the printability index. Inks BG and aIG show a significantly lower U_s_ further proving the higher shape fidelity of the printed strand and their low coalescence in comparison to ink rIG with also a lower standard deviation. Ink rIG, printed with the same material throughput, showed a significant lack of dimensional stability along the cross-section of the printed structures. An ideal value of U_s_ = 0 would indicate no difference between the printed strand and the maximum arc of the cross-section (compare Figure 15). The value measured for ink rIG was almost 106 ± 0.04 µm, which is 2.7-fold higher than the value for ink aIG and 6.9-fold higher in comparison to ink BG. 

Based on the high difference between the pores of HA ink and the inconsistency of the extrusion process, no results were measurable for this ink. 

Analysis of the filament thickness (f_t_) and the filament distance before fusion (f_d_) was conducted to underline the previous mentioned results for U_s_ and P_r_. The f_t_ was significantly different for all used compositions. F_t_ of ink BG (407 ± 9 µm) resembled the inner diameter of the used nozzle (410 µm) within experimental error and therefore a high stability and good printability of the material without fusion could be confirmed. With 496 ± 10 µm ink aIG revealed higher values than the BG ink. This material still offers a high shape stability during printing, but it worsens after the printing process. Due to a lack of material coherence likely due the filler inert character towards the alginate matrix, the structure begins to lose its integrity after a certain period of time. F_t_ results for ink HA could not be obtained due to the high difference in extrudability and no reproducible results. The presented resolution tree image in Figure 8 is set as an example, as already mentioned for the grid structure, but it was not investigated further based on the previously given reasons. 

Ink rIG behaves very different to BG ink, with a f_t_ of 507 ± 14 µm, which further indicates the instability of this ink, as already observed for P_r_ and U_s_. The results describe a more liquid material without high shape stability. Nevertheless, all compositions (ink HA excluded) showed high reproducibility.

Samples BG and aIG reveal the same minimum distance before strands begin to come into contact without detectable difference. Unexpectedly, ink rIG fuses together only at a significantly lower distance of 713 ± 23 µm. This finding, however, is most likely connected to various experimental influences, such as ambient temperature, printbed temperature (which was not controlled) or small batch variations between printing samples. Those potential errors produced by external variables will be the focus of future research. Despite the difference between the inks, a high reproducibility of each material could still be achieved, which is visible by considering the low standard deviations of the determined parameters. 

**Figure 9:**
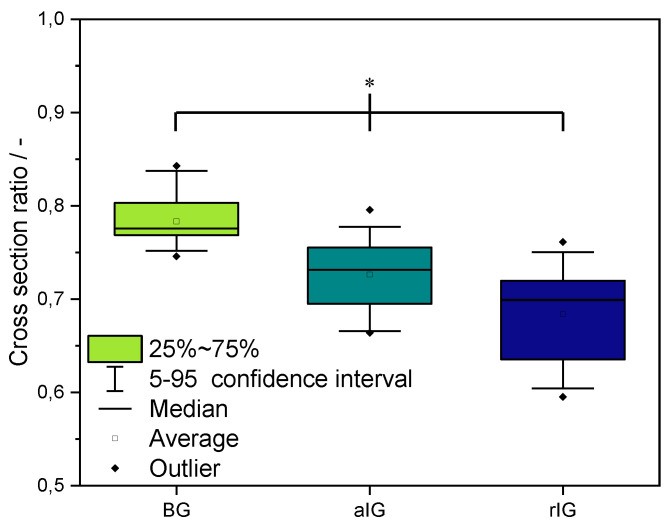
Analysis of the cross-section ratio for printable alginate–matrix composites (inks BG, aIG and rIG). Statistical analysis using one-way ANOVA indicated a significant difference (* *p* < 0.05) for all samples.

The cross-section ratio was calculated as described in Figure 16, for a more detailed analysis of the stackability of the strands. The results are shown in Figure 9. Here, a value of “1” represents perfect stackability and therefore no merging of the grid intersections.

Ink BG showed the highest average and median value of cross-section ratio as well as the lowest standard deviation, followed by inks aIG and rIG, matching the results presented in Figure 8. Due to this finding, ink BG is deemed the most suitable composite ink for printing multilayered 3D scaffolds. Ink aIG also led to promising results. Ink rIG only provides limited suitability for printing applications, due to the increased standard deviation and low cross-section ratio.

### 2.8. In Vitro Biocompatibility Studies

For the evaluation of the developed biomaterial ink regarding potential cytotoxic effects, preliminary tests using MC3T3-E1 cells were performed. Cell tests were, as already mentioned, only performed on BG ink, due to the inert character of the un-reactive glasses and the impossibility of generating 3D interconnected scaffolds with HA filler in the used ink composition.

**Figure 10:**
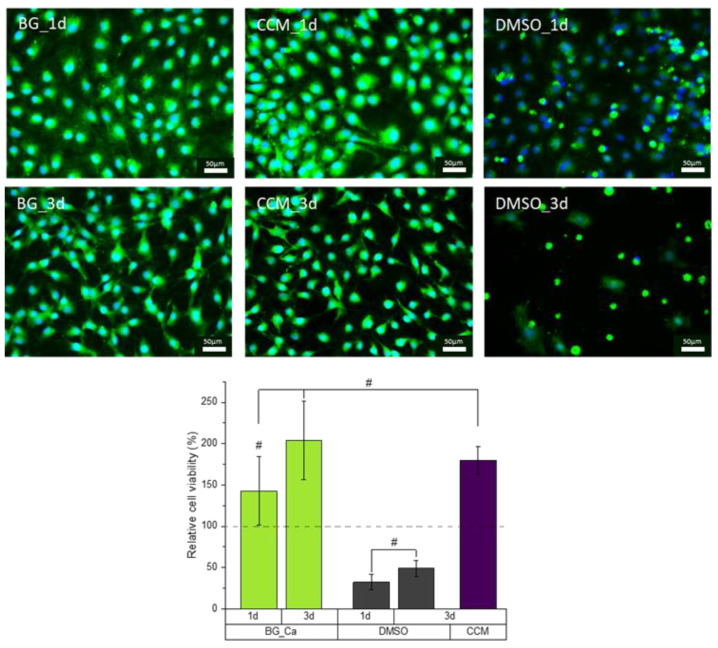
Fluorescence images of living cells (green) and cell nuclei (blue) of MC3T3-E1 cells cultured for 1 (**top**) and 3 (**middle**) days in CCM (positive control), 6% DMSO (negative control) and conditioned CCM of composites containing 30 *w/v* % BG. Relative cell viability (WST-8) of MC3T3-E1 cells (**bottom**) cultured for 1 and 3 days. Statistical analysis using one-way ANOVA indicated a significant difference (*p* < 0.05) for all samples. Samples labeled with # are found to be an exception.

Cells were incubated for 1 and 3 days in CCM containing the dissolution products of the BG samples. As demonstrated in the fluorescence images of Figure 10top, seeded MC3T3-E1 cells showed no significant change in their morphological expression, being in contact with the conditioned CCM in comparison to cells incubated in pure CCM (positive control). Observations 3 days after seeding (Figure 10middle) still do not indicate any impact of the dissolution products on the cells. Both proliferation and cell morphology are comparable to the positive control and show a much higher spread morphology and proliferation than the negative control (6% DMSO). 

Additionally, cell viability tests using the WST-8 assay were performed (Figure 10bottom). Results were calculated relative to the measured viability of the positive control in (%) 1 day post cell-seeding. As the fluorescence images already indicated, the viability of cells was not negatively influenced by the conditioned CCM. Cells in contact with the dissolution products even result in an increase in cell viability after 1 day. This positive effect on the cell viability further increased with an increase in incubation time. Here, the measured relative viability after 3 d was two-fold higher than the 1 d positive control with even a 20% higher viability compared to the positive control 3 days post cell-seeding. 

**Figure 11:**
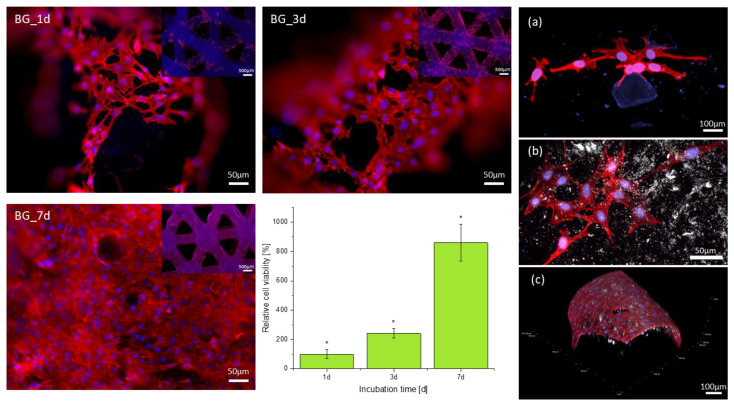
Fluorescence images of MC3T3-E1 cells directly seeded onto the BG–alginate composite scaffold surface. Images taken 1 d, 3 d and 7 d post cell-seeding. Cell viability test (WST-8) after 1d, 3d, and 7d post cell-seeding in relation to the cell viability after 1d post cell-seeding, set as reference (**bottom middle**). Confocal fluorescence microscopy images of scaffolds 1 d (**a**,**b**) and 7 d (**c**) post cell-seeding. MC3T3-E1 cells are observed with stained actin filament (red) and cell nuclei (blue). Statistical analysis using one-way ANOVA indicated a significant difference (* *p* < 0.05) for all time points of culture.

To support the results showing a positive influence of the material on cell viability and proliferation in indirect tests, tests with MC3T3-E1 cells directly seeded onto the 3D printed scaffolds were also carried out. Fluorescence images (Figure 11) taken after 1, 3 and 7 days post cell-seeding demonstrate adhesion and proliferation of the cells on the material surface for all periods of culture. After 1 day of incubation, cells showed already a widely spread and elongated morphology indicating promising cell conditions. A total of 3 days after seeding the number of cells increased, which demonstrates the proliferation ability of the adherent cells. After 7 days of incubation, the scaffold was completely covered with cells. Every visible printed strand was covered with cells building a dense multi-layer. Cells showed multiple physical contact points to neighboring cells already after 24 h, which increased over time. These findings are supported by confocal fluorescence microscopy images taken 1 day after cell seeding (Figure 11a,b). Cells exhibit several long fibers on the material surface which connect to neighboring cells (Figure 11b). Moreover, confocal microscopy images indicate the direct attachment of the MC3T3-E1 cells to the BG particle surface, proving the positive effect of this filler on cell attachment, leading also to a strong connection between cells (Figure 11a). Further, confocal fluorescence microscopy revealed a 3D growth of MC3T3-E1 cells not only on the top of the printed strands but also around their sides, covering the whole strand (Figure 11c).

Additional cell viability measurements using WST-8 assay (Figure 11bottom) were carried out for direct cell tests at the same culture times. Viability was calculated relative to the measured optical density (OD) after 1 day and for further comparison adapted to 100%. Results prove an increase in viability over time for the used MC3T3-E1 cells. After 3 days of culturing, the measured cell viability was already more than two-fold higher than after one day, supporting the fluorescence results indicating a higher cell number. This trend continued to 7 days of culture with an eight-fold higher relative viability compared to 1 day and a significant difference between every time point.

## 3. Discussion

This study investigated the 3D printing of alginate-based scaffolds using composite inks containing different filler types. The analysis of SEM images revealed not only the geometry but also the size distribution of the used filler particles. This knowledge is necessary to validate all following discussions. Large differences in particle size or very broad size distributions could not only lead to nozzle clogging during printing but could also induce undesired particle–particle interactions in the ink. Furthermore, it is widely known that large aspect ratios of fillers in composite inks (e.g., fibers as filler material) reduce the necessary volumetric infill for percolation and network development [44]. 

The analysis of at least 50 particles for each filler material revealed average particle sizes between 34 ± 12 μm and 72 ± 21 μm. This finding underlined the effectivity of the used sieving process as well as the suitability of the used particle–needle combination. Furthermore, other potential influences, such as aspect ratio differences, can be neglected for the upcoming discussion. 

Percolation plays a major role in the development of innovative polymer composites. In the case of the used particle-filled inks in this study, a percolation phenomenon would mainly influence the visco–elastic material properties during rheological testing and the final printing results. However, the obtained values of volumetric infill do not exceed typical critical values found in literature and therefore percolation effects are highly unlikely. Here, the threshold for percolation of spherical particles ranges from 0.16 (neighboring hard-core spheres [45]) to 0.29 (interpenetrating soft-core spheres [46]). On closer inspection of the SEM images, however, HA particles did show additional surface roughness. This surface roughness led to a 68-fold increase in the effective surface area for the used HA particles in comparison to the estimated value. Therefore, it can be concluded that potential improvements of printability and effects on rheological properties are not directly correlated to percolation but are linked to the increase in internal friction or particle–matrix interactions due to particle roughness. 

As shown from SEM analysis, the differences in rheological properties and printing results can be related to the shape and surface area of the particles and their respective ionic activity.

The rheological analysis revealed rheological elastic material behavior for aIG, BG and HA inks. On the other hand, rIG inks showed a crossover point from liquid to solid material behavior at about 1 rad/s. The performed frequency sweeps furthermore revealed comparable values of complex viscosity for aIG and BG inks. rIG ink showed the lowest value of complex viscosity in the whole tested regime, while HA ink exceeded the complex viscosity of BG ink by a factor of 10. 

All composite inks showed shear-thinning material behavior without a Newtonian plateau. These findings suggest a sufficient shape fidelity and stackability of structures printed with aIG, BG and HA inks, while rIG ink led to strand spreading. Furthermore, the observed material behaviors were confirmed by the measured material throughputs. Here, the throughput for equivalent pressures followed the fundamental behavior according to Hagen–Poiseuille [47], where the material throughput in a capillary decreases with increasing viscosity (for constant pressure and capillary geometry). However, the significantly increased viscosity of HA ink led to problems in combination with the used pneumatic extrusion system. At 240 kPa of extrusion pressure, the material throughput reached a value comparable to BG and aIG inks. The standard deviation, however, exceeded 50% in the 1 min experiments. This finding suggests only limited usability of the tested HA ink composition for 3D printing, since constant and reproducible material throughputs are necessary for artifact free printing results. Furthermore, from this section of the experiments, a good printability and stackability for aIG and BG inks was anticipated, while rIG ink might tend towards strand spreading and merging of intersections. Furthermore, the excellent reproducibility of extrusion experiments underlines the potential strand homogeneity during printing with aIG and BG inks.

The aforementioned predictions mostly held true for the printing experiments. BG and aIG inks showed nearly perfect printability with P_r_ indices of 1.1, while rIG ink only performed marginally worse with an index of 1.17. However, the longer standard deviation for rIG ink led to the conclusion that single pores differ more compared to the structures made using BG and aIG inks. This can be correlated to the predicted spreading property from the rheological analysis and is further underlined by the significantly worse uniformity of rIG compared to BG and aIG inks. Surprisingly, the uniformity did also reveal differences between BG and aIG inks that were not expected from previous experiments. Here, minor differences in rheological properties and reactivity are the only evident contrasts between BG and aIG inks, leading to an additional ionic crosslinking of alginate in the case of BG fillers. Schuhladen et al. [48] reported an increase in the release of Ca^2+^ ions of 13–93 BG powder in TRIS buffer in the tested time frame of three days. This release could already lead to a crosslinking reaction during the preparation of the composite ink and further explains the change in the BG ink rheological behavior in the first 24 h. Furthermore, ions bound to the surface of BG particles potentially strengthen the particle–polymer interactions after preparation of the ink. This additional crosslinking could still induce additional stability and therefore reduce spreading under load near the grid intersections. This phenomenon is also observable for the measured filament thicknesses. Here, the printing of BG composites resulted in strands with a thickness of 407 µm, which closely resembles the inner needle diameter of 410 µm. Both inert glass samples showed strand diameters of about 500 µm (496 µm for ink aIG and 507 µm for ink rIG), which still indicates a suitable printing quality. Since all printing experiments were performed with the same parameters (material throughput, printing velocity and Z-Offset), one can deduct that the differences in filament thickness are directly related to the inks spreading on the printbed and not to deviations in the printing setup. The differences of constructs made from aIG and rIG inks near cross-sections lead to further insights. Both the uniformity and cross-section ratio performance decreased when switching from inert angular to round particles. Here, the higher internal friction due to the increased geometrical hindrance and increased surface area of angular particles leads to more stable composite strand under load. This behavior was also observed for the flow analysis of powders with round and angular particles [49]. The increase in internal friction, which is usually achieved by alternating the polymer concentration of hydrogels, is widely known as the print performance enhancing factor [7]. Therefore, angular particles are recommended for improved printability at constant concentration of filler material. The difference, however, might only be noticeable in multilayer structures. HA ink was almost not printable, which was expected from the results of preliminary extrusion experiments. The high variation in extruded material led to incomplete strands and non-defined pores. For angular particles, reactivity only showed an influence on printing properties under load and might therefore only have an impact if scaffolds are printed in higher dimensions (height). Hence, the observed differences between rIG and HA inks must be correlated to the observed nano-surface-roughness of HA particles (see Figure 1, SEM images). The drastically increased internal friction in combination with possible ionic crosslinking led to a highly viscous composite ink that was not usable with the available printing set-up. Even though the pre-analysis predicted a good shape fidelity and stackability for HA inks, one can only speculate about the applicability of this ink with other printing set-ups. To the authors knowledge, this correlation of effective filler surface area, their geometry and printing performance, as well as the filler rheological influence, have not been described in the literature yet for composite inks. 

**Figure 12:**
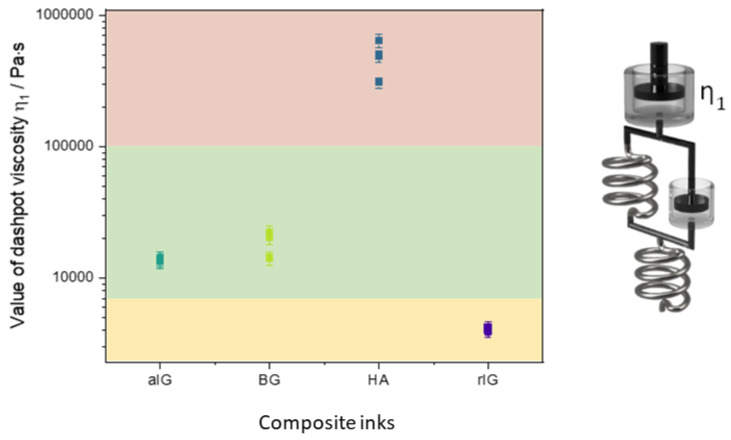
Illustration of the resulting windows of processability according to η_1_ obtained by the augmented Burgers model for the different inks investigated.

Figure 12 illustrates the core insight from the rheological modelling performed in this study. Here, the Maxwell dampener $\eta_{1}$ was identified as key parameter to successfully correlate strand spreading and stackability to the rheological analysis. Combining the experimental data from printing experiments with modelling results, one can define three windows of processability. For high values of η_1_, homogenous extruding was not possible with the used 3D printing setup (Figure 12, red). Samples with the lowest η_1_ value showed insufficient stackability and are therefore limited in their usability. This could be prevented by additional processing methods, such as in-print-crosslinking (Figure 12, orange) [50]. These boundaries define also the desired values for η_1_ to ensure sufficient shape fidelity and stackability (Figure 12, green). The general validity of this finding is to be determined and will be in the focus of future research using other types of inks. The generation of more relevant data will allow better definition of process window boundaries. 

The observed increase in spring moduli for the HA composite ink indicates a drastically stiffer and more elastic material behavior, which could be beneficial for load bearing scaffolds and cell types that favor stiff substrates. This is the case for mesenchymal stem cells, where an increase in the substrate stiffness leads to an increase in cell adhesion and a more spread morphology of these cells [51]. Further, the generated 3D printed scaffolds with an interconnected pore structure confirmed the already expected excellent printability of the BG composite ink. Stable and parallel strands were printed without collapse of the pores or merging of the different layers, even after a printing height of about 3 mm. Furthermore, the top layer of the scaffolds showed still high printing accuracy, with a strand diameter of about 430 µm after crosslinking, being in excellent agreement with the 2D results and the inner diameter of the used nozzle.

In addition, the performed preliminary in vitro cell test proved the suitability of the BG composite material for potential application in bone regeneration. The positive effect of bioactive glasses on cell behavior has been frequently reported in the literature. For example, Valerio et al. [52] reported an increase in osteoblast cell viability when in contact with BG60S bioactive glass compared to the biphasic calcium phosphate reference. Additionally, Luo et al. [29] showed an improvement in cell adhesion and rBMSC differentiation when in contact with 13–93 BG. The positive influence of 13–93 BG on the proliferation and differentiation of MC3T3-E1 cells was proven by Fu et al. [53] culturing MC3T3-E1 cells on 13–93 foam-replica scaffolds for 6 days, showing proliferation not only on the scaffolds surface but also inside the pore structure. The positive effect of 13–93 bioactive glass was also detectable in the performed indirect and direct biocompatibility tests in this study using MC3T3-E1 cells. In both cases, widespread cell morphologies were reported (visible in fluorescence images) with improved viability compared to the positive reference after every culturing time point. Further, cells showed a high rate of proliferation also on deeper printed layers with good cell–cell and cell–matrix interaction also on the sides of the printed strands covering them completely.

## 4. Materials and Methods

### 4.1. Biomaterial Ink Reperation

The polymer–glass/ceramic composite was prepared by heating phosphate buffered solution (PBS, Gibco, MA, USA) to 80 °C and adding 6 *w*/*v* % of polyvinyl-alcohol (PVA, 98% hydrolyzed, M_w_ 30,000 g/mol, Merk KGaA, Darmstadt, Germany) under constant stirring for 30 min. After complete dissolution of PVA, the solution was cooled to 37 °C and a mixture of filler (30 *w*/*v* %) and 10 *w*/*v* % alginate powder (Alg, PH176, VIVAPHARM^®®^ JRS PHARMA GmbH & Co. KG, Rosenberg, Germany) was added. This composition is set as a reference, since in preliminary studies (not shown here), bioactive glass (BG) as filler showed the best printing results regarding 3D structure and interconnective pore system. Due to BG high reactivity, the most extensive interaction between filler and matrix was expected for this composite. Therefore, preliminary studies with different ink compositions were carried out (results not shown) indicating the mentioned ink composition as the one leading to the best printing results. 

Four inorganic particle types were used as filler. As reactive fillers either round hydroxyapatite particles (HA, Medical Group Implant Coating Experts, Saint-Priest, France) or angular BG particles (13-93 BG composition, in wt %: 53% SiO_2_, 6% Na_2_O, 12% K_2_O, 5% MgO, 20% CaO, 4% P_2_O_5_ [54]) were used. As un-reactive, inert filler component either round inert glass particles (soda-lime glass, rIG, composition in wt%: 65% SiO_2_, 14% Na_2_O, 2.5% MgO, 8% CaO, 0.5–2% Al_2_O_3_, <0.15% Fe_2_O_3_, Strahlofix-kontrasil, Müller GmbH, Bayreuth, Germany) or angular inert glass particles (aIG) of comparable composition were used. For producing aIG particles, microscopy slides without frosted edges (Carl Roth, Karlsruhe, Germany) were used. BG and aIG had to be broken down using a jaw-crusher (Retsch, Düsseldorf, Germany). 

To obtain comparable particle size distributions, fillers were sieved to a particle size in the range 40–71 µm. Irsen et al. [55] and Seitz et al. [56] already reported a good printability of µm-sized particles (below 100 µm). 

After stirring at high speed to incorporate the dry components of filler and Alg to the PVA solution, the mixture was further homogenized by hand for at least 10 min to guarantee a homogeneous distribution of the filler components and create a smooth paste. 

After transferring the inks to a printing cartridge and centrifugation at 1200 rcf for 7 min (to remove entrapped air) the materials were stored overnight at RT in order to achieve the highest possible printability (influence of storage time shown in the results section). 

For material printing and extrusion, a 3D printer (BioScaffolder 3.1, GeSiM mbH, Radeberg, Germany) equipped with a printing nozzle of 22 G (410 µm inner diameter) was used. For an optimal printing result, the distance between the needle and the print surface was optimized (by preliminary experiments) so that the leading edge of the flow was in line with the needle. The pneumatic-based printer offers a printing head, which is movable in three room axes and a static printing platform. During all experiments, the printing velocity was kept constant at 10 mm/s and the pressure at 240 kPa, unless otherwise stated. 

### 4.2. Characterization

#### 4.2.1. Particle Size Distribution

Prior to the evaluation of the different filler particles on the printability of the ink, pure particles were analyzed using Scanning Electron Microscopy (SEM, Auriga, Zeiss, Jena, Germany) to describe particle morphologies and surface structures as well as the particle size distribution. Measurements were conducted on at least 50 particles using ImageJ software. 

#### 4.2.2. Filler Density and Volumetric Infill

The filler particle density ($\rho_{Filler}$) was determined using the Archimedes’ principle. The particulate samples were weighted in air and afterwards in ethanol. From the difference in masses, one can calculate the displaced volume and finally the density of the used fillers using Formula (1):

(1) $$\rho_{Filler}=\frac{m_{Air}\cdot \rho_{EtOH}}{m_{Air}-m_{EtOH}}$$

with

$m_{Air}$ = Sample mass in air

$m_{EtOH}\;$= Sample mass in ethanol

$\rho_{EtOH}$ = Density of ethanol (789 kg/m^3^)

Three separate measurements were performed for every filler type (*n* = 3). From the determined filler densities and the known mass of the filler particles and the used hydrogel (see Section 4.1 “Biomaterial ink preparation”) one can easily access the volumetric infill as follows (Formula (2)):

(2) $$V_{Filler}=\frac{m_{Filler}}{\rho_{Filler}}\;\;\;\;\;\;\;V_{total}=V_{Filler}+V_{Hydrogel}\;\;\;\;\;\;\;V\%=\frac{V_{Filler}}{V_{Total}}$$

with

$V_{i}$ = Volume (index)

$m_{i}$ = Mass (index)

$\rho_{i}$ = Density (index)

#### 4.2.3. Specific Surface Area Determination

To determine the specific surface area and possible pores on the surface of the particles which could influence the printing performance and the rheological behavior, nitrogen sorption analysis was performed. Measurements were conducted using a Micromeritics porosimeter (ASAP2460, Micrometrics Instrument, Norcross, GE, USA). The measurement was carried out twice.

#### 4.2.4. Material Throughput

Material throughput (MT) was analyzed to investigate the influence of the different particles on the amount of material extruded over time at RT. All inks with different filler particles incorporated were extruded for one minute and the weight of the extruded material analyzed using a high-resolution analytical balance (A&D GX-600, Mettler Toledo, Gießen, Germany). Additionally, the necessary pressures to obtain the same throughput as for the reference material composition (BG particles) were determined. For material throughput, *n* = 6 samples were measured after 1 min of extrusion.

#### 4.2.5. Biomaterials’ Printing Performance

To investigate the influence of filler particle shapes and their reactivity on the printing performance of the composite material, grids and resolution trees were generated. These were further investigated regarding the materials’ printability, shape fidelity, uniformity of strand diameter, filament thickness, filament distance and the cross-section ratio. For better comparability of the results, grids and resolution trees were generated with the same material throughput over time, based on the obtained results (reported in the “Material throughput” section). As a reference the throughput of the BG-based composite was used. All light microscopy images were taken within the first three minutes after the printing process with an invert light microscope (Primo Vert, Zeiss, Jena, Germany) and further analyzed with the ImageJ-Fiji software.

#### 4.2.6. Printability and Shape Fidelity (Semi-Quantification)

For investigation of the printing accuracy and the influence of the particles on this parameter, the printability, uniformity, cross-section ratio, filament thickness and minimum filament distance (better known as filament fusion test [57]) were investigated.

For the first three factors an orthogonal grid was printed, with 13 strands per layer and two layers in total. Two layers are reported to be suitable structures for qualitative and quantitative assessment of the material performance [36]. 

**Figure 13:**
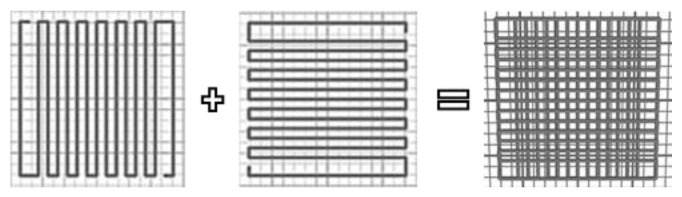
Top-view of the schematic printed layers and the resulting grid. Strand distance 1.8 mm, strand height 0.4 mm in a printing angle change of 90° per layer.

The strand distance was set to 1.8 mm and the strand height to 0.4 mm. The inner strand angles were changed between 0° and 90° to obtain perfect rectangular pores. The schematic top-view structure of the generated grid (right) and the two different layer orientations (left (0°), middle (90°)) are shown in Figure 13. The images were created with the software of the used printer. 

To avoid failures of strand deposition based on the printing process itself, no single strands were printed, but each layer was constructed using the function of meandering with an additional strand on each side (not considered for results evaluation). 

**Figure 14:**
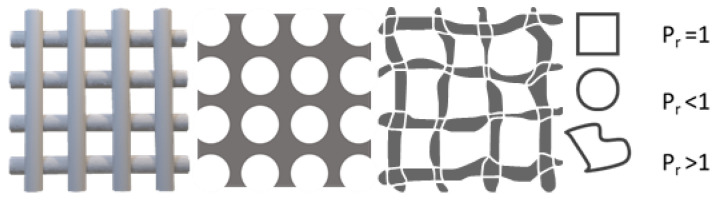
The influence of pore shape on the P_r_ index in a grid structure. The schematic structure was designed based on Ouyang et al. [35].

For evaluation, microscopy images were taken for *n* = 5 samples. Based on the images, the pore geometry was calculated on at least 40 randomly chosen pores, using the “Printability index” (*P_r_*) as a parameter for the interchannel pattern of flat 2D printed structures (Figure 14). Where *P_r_* = 1 means a perfect square shaped pore. For calculation of *P_r_*, Equation (3) was used [35]:

(3) $$P_{r}==\frac{L^{2}}{16A}$$

with

*L* = perimeter, *A* = area. 

#### 4.2.7. Uniformity of Strand Diameter (*U_s_*)

**Figure 15:**
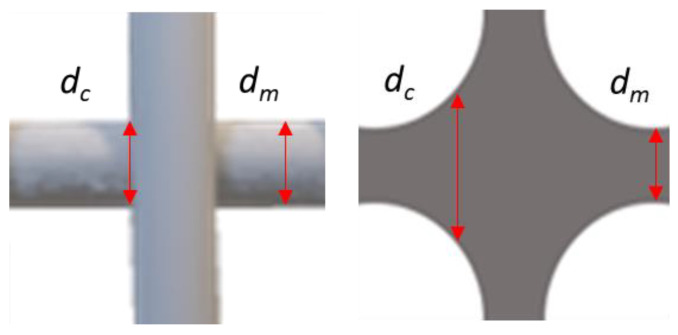
Schematic structure of the cross-section of two layers with high stability without any coalesce (**left**) and with poor integrity and coalesce of the strands (**right**). Equation (4) for calculation of the *U_s_* [36].

To identify the *U_s_* in the cross-section of the two printed layers, the diameter of at least 30 strands per composition was measured at the middle of the strand and the maximum arc at the cross-section of merging, as mentioned by Schwab et al. [36]. The diameter of the strand middle was then subtracted from the one of the maximum arc, following Equation (4). In this assessment, the closer the difference is to 0, the more congruent are the diameters and the higher is their uniformity. This indicates a high shape fidelity of the strands and good stackability of layers without merging. Materials with poor stability show more round-shaped pores due to the loss of strand integrity and merging to the previous layer and as a result of it a higher value of uniformity is achieved, as shown in the illustration in Figure 15.

(4) $$U_{s}=d_{c}-d_{m}$$

#### 4.2.8. Cross-Section Ratio

**Figure 16:**
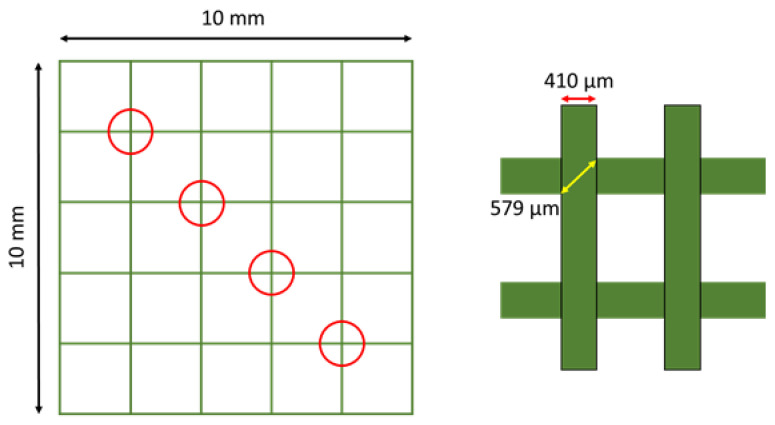
Schematic description of the cross-section ratio.

For the determination of stackability of hydrogel strands, the cross-section ratio was determined. The diagonal length of 5 cross-sections per image, resulting in 25 cross-sections, was measured in both directions and compared to the theoretical value. This theoretical value is defined as the diagonal length of a square with a side length equal to the used nozzle diameter (see Figure 16, right). The procedure was repeated for three individual pictures for each tested composite. The cross-section value is then calculated by dividing the theoretical value (579 μm for the used nozzle) by the measured distance.

#### 4.2.9. Filament Thickness (f_t_) and Filament Distance (f_d_)

**Figure 17:**
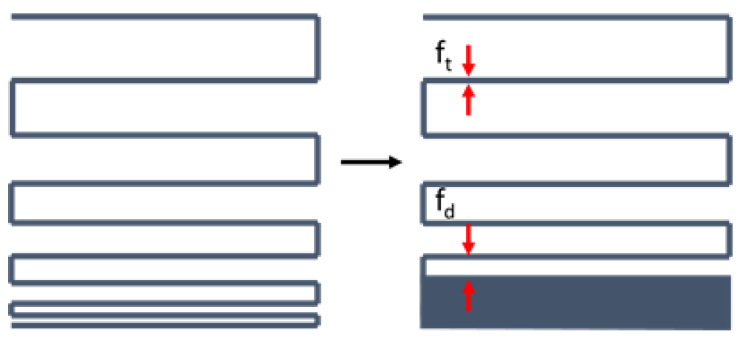
Single layered, meandered resolution tree for investigation of filament thickness (f_t_) and filament distance (f_d_).

For analysis of f_t_ and f_d_, a meandered single layer (resolution tree) was printed, with reduced strand distance after every change of direction (Figure 17) for further analysis of the strand stability and to compare the inks [57]. For strand homogeneity investigation, f_t_ of the printed strands was measured on *n* = 20 different sections. Further, f_d_, measuring the gap from the filament center to the center before the strands touch at only a single point, was measured on *n* = 10 sections.

#### 4.2.10. Dynamic Mechanical Analysis

For the dynamic mechanical analysis, a DHR-3 rheometer (TA Instruments, Newcastle, USA) equipped with a 40 mm plate–plate geometry (and counter geometry) was used. Additionally, a Peltier device was installed to ensure temperature control. A solvent trap, filled with deionized water, reduced sample drying and solvent evaporation to a minimum. The measurement gap was fixed at 500 μm with a trim gap offset of 25 μm. The standard measurement setup consisted of two basic measurement steps and was performed in triplicate using a fresh sample for every iteration:

(1) Frequency sweep: frequency sweeps were performed in a frequency range from 0.1 to 100 rad/s. An amplitude of 1% was used and deemed suitable by the following amplitude sweep.
(2) Amplitude sweep: a rest time of 120 s was set to ensure complete material relaxation. The measurement frequency was set to 10 rad/s and the amplitude interval ranged from 0.01 to 100%. Therefore, the end of linear viscoelastic regime was determined and the used amplitude for the previous frequency sweep was validated.

Additionally, due to the expected high reactivity of BG filler, and the necessity to adapt the materials printing pressure with increased printing time (not required for the other used filler particles), in preliminary printing studies, frequency sweeps for six time intervals were performed on one sample to determine potential crosslinking over time. All measurements were performed at room temperature (25 °C), regulated by the Peltier element, to ensure close correlation to the printing results. 

#### 4.2.11. Rheological Modelling

To generate further insights and reveal more detailed correlations between rheological properties and printing results, the modeling approach by Schrüfer et al. [42] was used. As a first step, the most suitable model system (out of commonly used rheological models) must be determined. The resulting adjusted R^2^ values for all samples were compared. Subsequently, one can calculate the respective model parameter values, as described elsewhere [42]. A self-written python script using the open-source statsmodels module (V. 0.13.0, further information on www.statsmodels.org, accessed on 15 September 2021) was used. Results were further validated randomly by the Statistika (StatSoft Europe, Hamburg, Germany) software package. 

#### 4.2.12. Scaffold Fabrication

**Figure 18:**
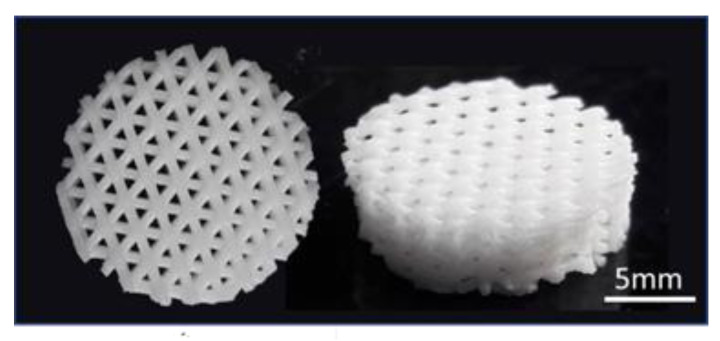
3D printed scaffolds of BG-alginate composites for direct cell biological characterization with a height of 2.8 mm and a diameter of 15 mm with an inter-layer angle change from 0° to 60° and interconnected pore system. Top view (**left**), side view (**right**).

For the direct in vitro biocompatibility test (described in the following section), 3D scaffolds were printed with 7 layers, 2.8 mm total height, a strand distance of 1.15 mm, an angle change from 0° to 60°, with every layer leading to an interconnected pore structure. After printing, scaffolds were crosslinked overnight in an aqueous solution of 0.1 M calcium-chloride dihydrate (CaCl_2_·2H_2_O, VWR chemicals, Darmstadt, Germany). After crosslinking samples were cut using a punching tool to a uniform size of 15 mm in diameter as shown in Figure 18. In total, *n* = 15 scaffolds were printed and divided evenly for the cell-culture tests.

#### 4.2.13. In Vitro Biocompatibility Studies

To prove the materials’ biological suitability for bone regeneration in non-load bearing areas, biocompatibility tests were performed with 3D printed structures. Due to the poor printing performance of the composition using HA particles as filler, (as shown in the “Results” section) and the inert character of the IG particles, in vitro tests were only performed with BG composites.

Preliminary cell tests were carried out with pre-osteoblast cells (MC3T3-E1, supplied by the Leibnitz-Institute DSMZ GmbH, Germany), being an established cell line often used in research in the field of bone regeneration [58,59]. Cells were cultured in cell culture medium (CCM, α-MEM + supplemented with 10% fetal calf serum, 1% penicillin and streptomycin and 1% L-Glutamine) at 37 °C, 95% humidity and 5% CO_2_. CCM was changed every 3–4 days.

Indirect and direct cell tests were performed in 2 mL of CCM. For the indirect test, 100,000 cells were seeded onto the surface of a multi well cell-culture plate only being in contact with CCM containing the dissolution products of the material, without direct contact to the samples. For indirect tests, cells and samples were incubated separated from each other in CCM. After 24 h post-seeding, the CCM was removed from the cells and exchanged with the one containing the dissolution products of the samples. Additionally, cells were incubated with CCM as a positive reference and 6% (*v*/*v*) dimethyl sulfoxide (DMSO) as a negative reference. The cell viability was calculated in (%) with respect to the positive control after 1 day.

For the direct test, 200,000 cells were directly seeded onto the surface of the 3D printed scaffolds.

Cell tests were performed 1 or 3 days post cell-seeding to evaluate the materials cytocompatibility and cells’ ability to proliferate, before the CCM was changed for the first time. Direct cell tests were additionally performed after 7 d of incubation for further evaluation. Before cell seeding, scaffolds were washed twice with HBSS (Hank’s balanced salt solution, Gibco) for 5 min each, to remove surplus crosslinker. Afterwards samples were disinfected using a 5% (*v*/*v*) solution of penicillin and streptomycin in HBSS for 5 min, followed by a further washing step with HBSS for 5 min. The change in viability was calculated in (%) in relation to the results 1 d post cell-seeding. 

#### 4.2.14. Cell Viability

The biocompatibility of the samples containing BG particles as filler was determined using WST-8 assay (CCK-8, Sigma-Aldrich, Taufkirchen, Germany) evaluating the viability of the MC3T3-E1 cells. Tests were performed following the manufacturer’s protocol. A concentration of 5% (*v*/*v*) of WST-8 was used and either incubated for 1 h (indirect) or 4 h (direct tests) before the measurement in a plate reader (FLUOstar Omega, BMG LABTECH, Ortenberg, Germany) at 450 nm. For direct cell tests, samples were transferred to a new multi-well cell culture plate before WST-8 assay was performed. N = 3 samples were measured for each direct and indirect test. 

#### 4.2.15. Cell Morphology

Additionally, cell morphology analysis was performed for further investigation of the materials cytocompatibility for indirect tests and in direct contact with the scaffolds. Images were taken using a fluorescence microscope (Primo Vert Axio, Zeiss, Oberkochen, Germany). For indirect tests, live staining was performed with the green fluorescent dye calcein (calcein AM; Thermo Fisher, Erlangen, Germany) staining the cell membrane. For the direct tests, red rhodamine phalloidin (Thermo Fisher, Erlangen, Germany) was used to dye the actin filament of the cells. In both tests, cell nuclei were stained blue using 4′,6-diamidino-2-phenylindole (DAPI, Thermo Fisher, Erlangen, Germany). 

Cells were fixed on the samples using a fixing solution (pH 7.4, based on HBSS containing paraformaldehyde). All stainings were performed following the manufacturers’ protocols. To investigate the cell–material interaction in more detail, additional confocal fluorescence microscopy (Eclipse Ni-E equipped with an A1R HD Scan head, Nikon, Japan) was performed on 3D printed scaffolds, 1 d and 7 d post cell-seeding. N = 3 samples were analyzed for each direct and indirect test as well as for every investigated time period. 

## 5. Conclusions

The main focus of this work was to assess the influence of different particle shapes of fillers of different surface reactivity on the printability of alginate-based composite inks. The results indicated a great impact of the effective filler surface area on the rheological properties and therefore also on the material’s printing behavior. Angular BG and inert glass containing inks showed the same viscosity within experimental error and no appearance of an influence of the particles’ surface reactivity on this parameter. The surface reactivity of fillers regarding the particle–matrix interaction was shown to play only a role in determining the stability of the printed strands as demonstrated during the measurement of the filament thickness. In general, with angular fillers, high levels of accuracy could be reached.

As another important influencing factor, the surface topography of the filler particles was determined. Inks with smooth, round inert glassy filler particles showed a significant minor impact on the rheological properties, especially at lower frequencies, compared to angular particles. This effect leads to a limited printing performance if more than one layer should be generated. Surprisingly, high viscosity values were reached for HA inks probably based on the nano surface roughness of HA particles. Although the ink incorporating HA was not reproducibly printable and showed an inhomogeneous behavior, preliminary results indicate its printing potential if no nozzle clogging occurs, implying a promising printability with a lower filler content. 

It can be concluded that the highest impact of filler particles is based on their shape, with further influence of the particles’ surface topography and surface area. Reactivity with the surrounding matrix is especially important for the stability of the printed object with only minor or no influence on the materials rheological profile or performance during the printing process itself. Results further indicate that a higher filler content in the composite can be achieved if round particles with a smooth surface are used, while excellent printing results can be obtained with particles of a higher effective surface area at lower filler contents. Further, in preliminary cell studies using MC3T3-E1 cells, the positive impact of the composite containing BG regarding cell adhesion, proliferation and viability could be shown in indirect and direct tests.

For future research, the influence of the surface topography of the filler particles should be considered in detail. Furthermore, tests including particle size analysis will be conducted to investigate how smaller (nm) and larger particles (higher µm scale) influence the materials’ printability. In addition, the combination of angular articles and round particles with and without a surface structure should be considered to identify the dominating factor in 3D printing experiments.

## Figures and Tables

**Table 1:** Median values with standard deviation and Y–X ratio for the measured filler particles. Values are shown in (µm).

Sample	X Dimension	Y Dimension	Y–X Ratio
aIG	34 ± 12	63 ± 21	1.87
BG	41 ± 17	72 ± 21	1.76
HA	49 ± 12	49 ± 12	0.99
rIG	65 ± 12	64 ± 12	0.98

**Table 2:** Filler densities with standard deviation and calculated volumetric infill.

Filler Name	Filler Density (g/cm^3^)	Volumetric Infill	Estimated Surface Area (mm^2^/mL)
aIG	2.34 ± 0.03	0.11	15,308
rIG	2.46 ± 0.01	0.11	5701
HA	3.05 ± 0.03	0.09	6028
BG	2.59 ± 0.02	0.10	11,356

**Table 3:** Comparison of estimated and real surface area for HA particles.

Filler Name	Surface Area per Gram (m^2^/g)	Surface Area per mL (mm^2^/mL)
HA (solid approximation)	0.02	6028
HA (nitrogen sorption)	1.38	412,560

**Table 4:** Resulting adjusted R^2^ for commonly used rheological models.

Sample ID	Maxwell	Kelvin-Voigt	Jeffreys	Zener	Burgers	Augmented Burgers
aIG	0.71	0.69	0.77	0.88	0.89	0.93
BG	0.70	0.70	0.76	0.89	0.88	0.93
HA	0.48	0.87	0.53	0.93	0.69	0.97
rIG	0.80	0.63	0.85	0.89	0.94	0.94

## Data Availability

The datasets generated during and/or analyzed during the current study are available from the corresponding author on reasonable request (A.R.B and R.D).
